# Management of Tennis Elbow with sodium hyaluronate periarticular injections

**DOI:** 10.1186/1758-2555-2-4

**Published:** 2010-02-02

**Authors:** Robert J Petrella, Anthony Cogliano, Joseph Decaria, Naem Mohamed, Robert Lee

**Affiliations:** 1 Dept Medicine, Canadian Centre for Activity and Aging, 801 Commissioners Road, London, N6C5J1, Canada; 2 Dept Medicine, Fowler-Kennedy Sport Medicine Clinic, 1490 Richmond St, London, N6C2 M3, Canada; 3 Dept Kinesiology, Canadian Centre for Activity and Aging, 801 Commissioners Road, London, N6C5J1, Canada; 4 Dept Family Medicine, 801 Commissioners Road, London, N6C5J1, Canada; 5 Sport Medicine, University of Waterloo, 1100 University Ave, Waterloo, Canada

## Abstract

**Objectives:**

To determine the efficacy and safety of peri-articular hyaluronic acid injections in chronic lateral epicondylosis (tennis elbow).

**Design:**

Prospective randomized clinical trial in primary care sport medicine.

**Patients:**

Three hundred and thirty one consecutive competitive racquette sport athletes with chronic (>3 months) lateral epicondylosis were administered 2 injections (first injection at baseline) into the subcutaneous tissue and muscle 1 cm. from the lateral epicondyle toward the primary point of pain using a two-dimensional fanning technique. A second injection was administered 1 week later.

**Outcomes measures:**

Assessments were done at baseline, days 7, 14, 30, 90 and 356. Efficacy measures included patient's visual analogue scale (VAS) of pain at rest (0-100 mm) and following assessment of grip strength (0-100 mm). Grip strength was determined using a jamar hydraulic hand dynamometer. Other assessments included patients' global assessment of elbow injury (5 point categorical scale; 1 = no disability, 5 = maximal disability), patients' assessment of normal function/activity (5 point categorical scale), patients/physician satisfaction assessment (10 point categorical scale), time to return to pain-free and disability-free sport and adverse events as per WHO definition. Differences between groups were determined using an intent-to-treat ANOVA.

**Results:**

Average age of the study population was 49 years (± 12 years). One hundred and sixty-five patients were randomized to the HA and 166 were randomized to the control groups. The change in VAS pain was -6.7 (± 2.0) for HA vs -1.3 (± 1.5) for control (p < 0.001). The VAS post handgrip was -7.8 (± 1.3) vs +0.3 (± 2.0) (p < 0.001) which corresponded to a significant improvement in grip of 2.6 kg in the HA vs control groups (p < 0.01). Statistically significant improvement in patients' global assessment of elbow injury (p < 0.02), patients' assessment of normal function/activity (p < 0.05) and patients/physician satisfaction assessment (p < 0.05) were also observed favoring the HA group. Time to return to pain-free and disability-free sport was 18 (± 11) days in the HA group but was not achieved in the control group. VAS changes were maintained in the HA group at each followup while those in the control significantly declined from baseline. Assessment of patient and physician satisfaction continued to favor the HA group at subsequent followup.

**Conclusion:**

Peri-articular HA treatment for tennis elbow was significantly better than control in improving pain at rest and after maximal grip testing. Further, HA treatment was highly satisfactory by patients and physicians and resulted in better return to pain free sport compared to control.

## Background

Chronic tennis elbow or lateral epicondylosis produces symptoms of pain and functional disability. Typical treatments include RICE for acute exacerbations as well as oral or topical NSAIDs, bracing and physical therapy. However, there is no consensus on treatment while efficacy of existing treatments is poor. Intra-articular hyaluronic acid (HA) has shown efficacy equivalent to NSAID in the treatment of osteoarthritis while it's periarticular efficacy and safety have recently been reported for soft tissue use in acute ankle sprain. Hence, many patients, particularly those who require more rapid improvement to return to sport or work activity, or those in whom previous therapies have not achieved expected results, would benefit from a more rapid alleviation of symptoms, while still achieving the longer term benefits of hyaluronic acid that have been reported in other soft tissue indications.

Previous studies regarding treatment of chronic tennis elbow have shown lack of consensus as well as variable efficacy and high incidence of adverse effects [1]. Hyaluronic acid has been used in soft tissue application for acute ankle sprain with high degree of efficacy and very limited side effect. Hence, given the biocompatibility of HA in treatment of acute ankle sprain we may show efficacy in terms of pain and function with low incidence of side effect and treatment of chronic tennis elbow.

Tennis elbow (lateral epicondylosis), a common cause of chronic elbow pain and wrist extensor dysfunction in adults, affects 1-3% of the general population each year [2]. Tennis players have been reported to account for 5-8% of all cases, although between 40-50% of all tennis players will be afflicted with the condition at some time [3]. Lateral epicondylosis is most prevalent in the fourth decade of life and the syndrome is rarely seen in individuals under the age of 30. Localized tenderness around the lateral epicondyle generally characterizes the condition, and pain can be reproduced by resisted extension of the wrist or the middle finger with the elbow in a straight position. The injury results in elbow pain that usually heals spontaneously, although it can become a source of chronic pain and morbidity if left untreated. On average, a typical episode of lateral epicondylosis lasts 6-24 months [4]. The primary lesion and epicondylosis consists of micro ruptures and result in inflammatory granulation tissue in the tendinous portion of the origins of the forearm musculature just distal to the epicondyle of the humerus. The lesion is found primarily in the extensor carpi radialis brevis (ECRB) origin, with less frequent involvement of the extensor carpi radialis longus (ECRL) and the anterior portion of the extensor digitorum communis [5]. Nirschl [6] maintains that angiofibroblastic hyperplasia, resulting from avascular compromise and subsequent micro tears in the origin of the ECRB, is the basic cause of chronic lateral epicondylosis. While the muscle fibres have adequate blood supply and good healing potential, the tendon fibres attached to the periosteum are relatively avascular and are prone to ischemic stress and thus slow to heal [7]. Recent studies of chronic tennis elbow have not found any significant evidence of inflammatory processes and the term epicondylosis has been suggested as a more appropriate term than epicondylitis [8].

There is currently no consensus on the optimum treatment, but numerous options are available. The best available scientific evidence suggests that topical and possibly oral non-steroidal anti-inflammatory drugs may be the most useful for short-term pain relief. Corticosteroid injections may be beneficial as a temporary measure but carry the risk of possible adverse affects [9,10]. Symptoms usually start with an ache in the extensor aspect of the forearm with certain movements that become localized in the lateral epicondyle. Visible swelling is not a common feature in lateral epicondylosis and, if present, should suggest some other pathology. Elbow radiographs are usually normal and seldom helpful. Ectopic calcification of the lateral epicondyle appears in approximately 25% of the cases but its presence does not appear to alter prognosis. Differential diagnosis should include referred pain from the cervical spine, rheumatoid arthritis, radial tunnel syndrome and compression of the posterior interosseous nerve [11].

Hyaluronic acid is a natural occurring biological substance, which has been shown to have a positive effect in inter-articular administration for osteoarthritis [12] as well as more recently periarticular use in acute ankle sprain [13]. These studies have shown improved pain and functional range of the arthritic as well as the soft tissue injury with high degree of patient satisfaction and few adverse events. Previous studies of topical NSAID and botulinum toxin for the treatment of epilateral epicondylitis [14,15] have shown improved pain at 1-3 months post injection. However, this was associated with rash, mild gastric upset [14] and digit paresis and weakness of finger extension [15]. Further, efficacy was not assessed long term. Hence, given the lack of consensus, the high rate of adverse effects and lack of long term followup with current options, use of HA, which is relatively free of these adverse effects and has been used in ankle sprain with long term efficacy [16], could prove to be an option for patients with chronic tennis elbow.

Hyaluronic acid is an un-branched, high molecular weight polysaccharide distributed throughout the body, especially as a major component of synovial fluid cartilage and surrounding structures of arthroidial joints. The primary role of the HA in these tissues is to maintain their visco-elastic structural and functional characteristics. Given the long term efficacy and safety of periarticular HA in acute ankle sprain, it would suggest similar protective effects of HA in chronic lateral epicondylosis.

We are unaware of any other published studies that have prospectively followed patients who were administered HA in lateral epicondylosis for treatment of tennis elbow in clinical practice.

Hence, we hypothesized that HA administered in the soft tissue of lateral epicondylosis for chronic tennis elbow will be well-tolerated with few adverse events and result in improved clinical pain and function outcomes from baseline to long term followup compared to patients administered placebo.

## Methodology

**Figure 1:**
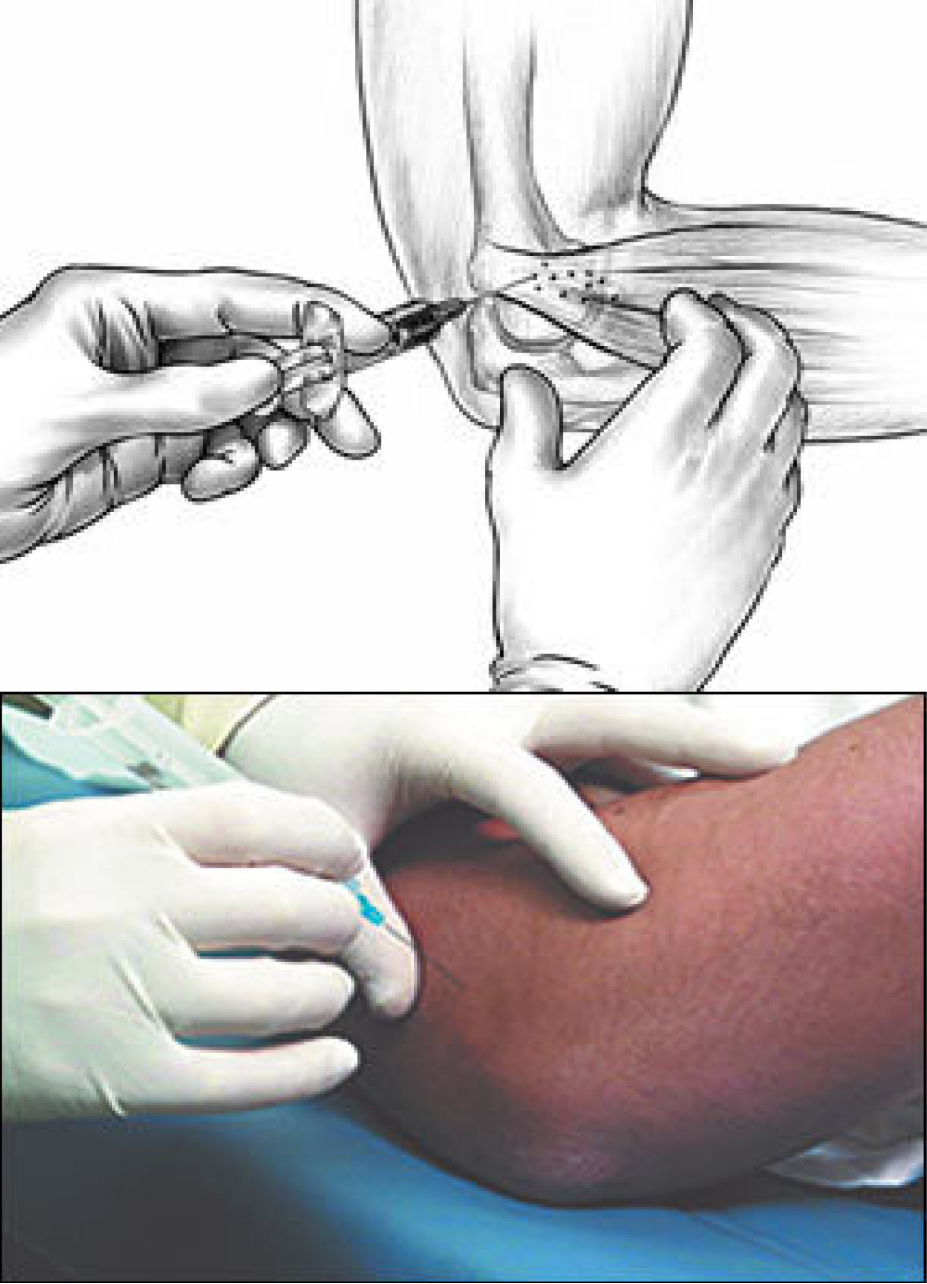
**Localization and administration of HA**.

We collected data on 331 consecutive patients administered HA (a clear solution of sterile 1% sodium hyaluronate in a phosphate buffered saline contained in a prefilled syringe; 1.2 cc) [165 patients] versus 166 patients administered 1.2 cc saline placebo. Treatment course was randomized and consisted of 2 injections (1 at baseline and a second at 7 days). Injections were administered using blinded syringes affixed to a 27-gauge, 1-inch needle. Skin was prepped using betadine 1%. Injections were delivered by the study physician using a standard approach along the lateral epicondyle with the affected arm flexed and resting on a firm surface. Injections were be administered into the soft tissue 1 cm from the lateral epicondyle at the point of greatest pain in two planes using a fanning technique [13] (Fig. 1) whereby contents were injected on withdrawl of the needle from the point of maximal tenderness in a single puncture. All patients had radiograph to exclude other pathologies at the discretion of the study physician (ie to exclude fracture). None took any other treatment modalities during the observation period or 48 hours prior to assessments. Assessments included general demographics, comorbidities and previous treatments. Patients rated pain on a 10 cm VAS, with 0 representing no pain and 10 representing maximal pain. Patients' global assessment of elbow injury (5 point categorical scale; 1 = no disability, 5 = maximal disability), patients' assessment of normal function/activity (5 point categorical scale; 1 = no change in function/activity, 5 = maximal change in normal function/activity) and physician's global assessment of elbow injury (5 point categorical scale; 1 = no impact of injury on function, 5 = maximal impact of injury on function) were also collected. Global assessments have not been validated but have been used previously by our and other groups to link the findings to implementation into routine practice. Time to return to pain and disability-free sport and adverse events were determined from review of a patient diary. After enrollment, patients were randomized (1:1) to one of two treatments using a computer-generated randomization schedule: HA or placebo.

Follow-up examinations were completed at Day 14 (± 2 days), Day 30 (± 2 days), Day 90 (± 2 days) and at Day 356 (± 7 days). Patients will assess pain on a VAS at rest and after assessment of grip strength. Grip strength will be determined using a jamar hydraulic hand dynamometer (Sammons Preston, Bolingbrook, Illinois). Assessment will be conducted with the patient's elbow fully extended, shoulder in neutral position and the dynamometer's handle in the middle position. Patients will perform three grip tests on the affected arm with a mean score calculated and used for analysis.

During the study, including the follow-up period, the patients received usual care including RICE (rest, ice, compression and elevation). Use of any analgesics was prohibited and all concomitant medication use was recorded in the patient's diary. Specifically, no NSAID, corticosteroid or topical analgesics were allowed during the study. ASA at the dose of 325 mg and less for cardiovascular prophylaxis was allowed.

Patients were assessed for pain on a VAS at rest and after assessment of grip strength. Patients' global assessment of elbow injury (5 point categorical scale), patients' assessment of normal function/activity (5 point categorical scale), and physician's global assessment of elbow injury (5 point categorical scale) was performed. Also, patients/physician satisfaction assessment (10 point categorical scale; 1 = no satisfaction with the procedure, 10 = very high satisfaction with the procedure) and review of a patient diary for adverse events and return to pain and disability-free sport was performed.

The primary outcome measures were an improvement on the VAS-pain at rest in the affected elbow and VAS-pain immediately following grip strength testing. Secondary outcome measures included patients' global assessment of elbow injury (5 point categorical scale), patients' assessment of normal function/activity (5 point categorical scale), physician's global assessment of elbow injury (5 point categorical scale), patients/physician satisfaction assessment (10 point categorical scale), time to return to pain and disability-free sport, concomitant medication use and adverse events.

The patients were recruited and screened in 4 sites (Canadian Center for Activity and Aging; University of Western Ontario-Fowler-Kennedy Sport Medicine Center; U Waterloo Sport Medicine Center; U Guelph Sport Medicine Center). Patients eligible for the study were 18 or older, with clinically or radiographic diagnosis of tennis elbow, and who were newly referred to the medical outpatient clinics at the author's institution which are primary sport medicine referral centers serving a population of 1.5 million patients. Inclusion criteria were pain at the lateral side of the elbow that had persisted more than 3 months and pain at the lateral epicondyle during resisted dorsiflexion of the wrist with the elbow in full extension. Exclusion criteria were previous local injection treatments (ie. corticosteroid injections or acupuncture), nerve entrapment or systemic neuromuscular disorders.

The diagram below provides a description of the overall study design (Table 1).

**Table 1:** Study Flow Chart

Evaluations	Baseline/Day1	Day 7	Day 14	Day 30	Day 90	Day 356 Day
Informed Consent	X					
Medical History	X					
Vital Signs and Physical Exam	X					
X-Ray Evaluation	X					
Patients VAS of pain at rest	X	X	X	X	X	X
Patients VAS of pain after grip assessment	X	X	X	X	X	X
Patients global assessment of elbow injury	X	X	X	X	X	X
Patients assessment of normal function/activity	X	X	X	X	X	X
Physician's global assessment of elbow injury	X	X	X	X	X	X
Patients/physician satisfaction assessment		X	X	X	X	X
Time to return to pain and disability-free sport			X	X	X	X
HA or placebo Administration	X	X				
Concomitant Medications	X	X	X	X	X	X
Adverse Events		X	X	X	X	X

The analyses of the efficacy endpoints used an intent-to-treat (ITT) population. The ITT population was patients who received at least 1 injection of double-blind study therapy. For the efficacy analysis, all patients were counted in the group they were randomized to irrespective of their actual treatment assignment.

### Statistical Power and Sample Size

For the primary analysis of the change from baseline in elbow pain we estimated a sample size of 160 per group using a 40% difference in resting VAS scores at Day 30 between treatment and control, a potential dropout rate of 25% and 95% confidence assuming a standard deviation of <10 mm of the mean deviation, an α of 5%, and a β level of 10%.

## Results

Both groups (HA = 165 vs placebo = 166) were similar for age (49 ± 15 vs 47 ± 11) and gender (55 vs 53% male) distribution (Table 2). There was also no difference among groups in the duration of their symptoms (26 vs 33 months). There were no serious adverse events reported throughout the study. Three patients (1.8%) in the HA and 5 patients (4%) in the control reported pain during injection. No other adverse events were reported. No subjects withdrew from the study during the treatment phase.

**Table 2:** Comparison of HA and control baseline, 30, 90 and 356 days followup.

	Baseline		D30		D90		D356	
	HA	Control	HA	Control	HA	Control	HA	Control
Age (y)	49 ± 15	47 ± 11						
Male %	55	53						
Duration of symptoms (m)	18 ± 17	22 ± 18						
VAS-rest (cm)	8.5 ± 11.1	8.4 ± 1.6	2.2 ± 1.2*	7.1 ± 1.3*@	2.5 ± 1.4*	6.7 ± 1.5*@	2.4 ± 1.4*	7.7 ± 1.3*@
VAS-grip (cm)	9.8 ± 1.1	9.6 ± 0.4	2.0 ± 1.5*	9.9 ± 1.3*@	2.2 ± 1.8*	9.3 ± 1.4*@	2.9 ± 1.4*	9.1 ± 1.1*@
PGS	0.3 ± 1.1	0.4 ± 1.1	4.6 ± 1.4*	1.6 ± 2.2*@	4.8 ± 0.6*	1.9 ± 0.3*@	4.8 ± 0.9*	1.1 ± 1.8*@
Grip (PSI)	49.2 ± 1.1	47.9 ± 0.4	68.0 ± 2.1	45.5 ± 1.1*@	67.7 ± 3.0*	48.1 ± 2.3*@	65.7 ± 1.8*	45.6 ± 1.3*@
PANF	1.1 ± 2.1	1.7 ± 2.2	4.4 ± 0.2*	2.6 ± 0.4@	4.8 ± 0.1*	1.3 ± 0.7*@	4.6 ± 0.3*	0.9 ± 1.9*@
PGA	1.1 ± 1.0	0.9 ± 1.2	4.3 ± 1.1*	1.8 ± 2.2*@	4.6 ± 1.1*	2.0 ± 1.7*@	4.7 ± 0.5*	1.3 ± 0.7*@
AE (N)			3	5				

VAS-pain are scored as a 100 mm VAS (0 = no pain; 100 = worse pain ever); grip strength (conducted with the patient's elbow fully extended and the dynamometer's handle in the middle position. Patients will perform three grip tests on the affected arm with a mean score calculated and used for analysis--measure in kg); PGS is patient global satisfaction using a 5 point categorical scale (0 = not satisfied, 5 = fully satisfied); PANF is patient assessment of normal function using a 5 point categorical scale (0 = no return to normal function, 5 = full return to normal function); PGA is physician's global assessment of elbow injury using a 5 point categorical scale (0 = poor patient elbow function and poor pain management, 5 = normal patient elbow function and normal pain management); AE are adverse events reported.

* = p < 0.05 (within groups); @ = p < 0.05 (between groups)

Mean baseline rest VAS was similar (8.5 ± 1.1 cm and 8.4 ± 1.6 cm) for HA and control respectively. VAS pain at rest and after grip testing was significantly better in the HA vs control (Table 2) at D 30. This was associated with significantly greater grip strength, patient global satisfaction and assessment of normal elbow function in the HA group vs control (Table 2). Physician global assessment of elbow injury was significantly better for the HA vs the control (Table 2). These differences persisted at each follow up assessment (90 and 356 days). Time to return to pain-free and disability-free sport was 18 (± 11) days in the HA group (in 147 patients; 89% response rate) but was not achieved in any of the control group patients.

## Discussion

There is currently no consensus in the management of chronic tennis elbow. Several, reviews have included various therapies targeting local or systemic interventions. Further, these therapies have not shown ability to shorten the disability period nor have they shown long term benefit. In our study, patients who received HA for lateral epicondylosis (tennis elbow) had significantly greater improvement in VAS pain at rest and after grip testing than control that persisted to 356 days followup. The treatment was highly satisfactory to patients and physicians and was associated with very few minor and transient adverse effects. Given the less than optimal treatment options for tennis elbow and given the associated chronic morbidity associated with this condition, periarticular injection with HA may provide an alternative for clinicians and their patients.

## Competing interests

The authors declare that they have no competing interests.

## Authors' contributions

RJP contributed to the study design, data collection, analysis and manuscript preparation.

AC contributed to the data collection.

JD contributed to the study design, data collection and manuscript preparation

NM contributed to the data collection.

RL contributed to the data collection.

All authors read and approved of the final manuscript.
